# Healthcare providers' perspectives on implementing a primary care–based initiative to increase equitable bowel cancer screening participation in Australia

**DOI:** 10.1093/heapro/daag152

**Published:** 2026-10-13

**Authors:** Joyce Jiang, Edwin Zou, Lanxi Huang, Jennifer McIntosh, Mark Jenkins, Carlene Wilson

**Affiliations:** Centre for Epidemiology and Biostatistics, Melbourne School of Population and Global Health, The University of Melbourne, Bouverie Street, Carlton, VIC 3053, Australia; Department of General Practice and Primary Care, The University of Melbourne, Grattan Street, Parkville, VIC 3010, Australia; Centre for Epidemiology and Biostatistics, Melbourne School of Population and Global Health, The University of Melbourne, Bouverie Street, Carlton, VIC 3053, Australia; Centre for Epidemiology and Biostatistics, Melbourne School of Population and Global Health, The University of Melbourne, Bouverie Street, Carlton, VIC 3053, Australia; Department of General Practice and Primary Care, The University of Melbourne, Grattan Street, Parkville, VIC 3010, Australia; Centre for Epidemiology and Biostatistics, Melbourne School of Population and Global Health, The University of Melbourne, Bouverie Street, Carlton, VIC 3053, Australia; Centre for Epidemiology and Biostatistics, Melbourne School of Population and Global Health, The University of Melbourne, Bouverie Street, Carlton, VIC 3053, Australia

**Keywords:** bowel cancer screening, health equity, ethnic minority populations, culturally and linguistically diverse communities, primary care, patient navigation, healthcare provider endorsement, qualitative research, Alternative Access Model

## Abstract

Bowel cancer is the fifth most common cancer in Australia and the second leading cause of cancer-related death. Although the National Bowel Cancer Screening Program mails test kits to eligible Australians, uptake remains suboptimal, particularly among underserved populations. The Alternative Access Model (AAM) is a primary care–based strategy through which healthcare providers (HCPs) distribute kits directly to eligible patients, enabling practitioner endorsement and patient navigation. This study explored HCPs’ perceptions of the acceptability, feasibility, barriers, and enablers of implementing the AAM in general practices serving ethnically and socioeconomically diverse populations. Semi-structured interviews were conducted with primary HCPs in Melbourne, Australia. Nine general practice clinics participated in a two-phase study. Thirty-seven interviews were conducted with general practitioners (*n* = 13), practice nurses (*n* = 15), and practice managers (*n* = 9). Data were analysed using NVivo 15. Most participants (89%) were unfamiliar with the AAM. Once explained, the model was perceived as acceptable because it aligns with primary care’s preventive role; however, implementation would be feasible only if barriers such as lack of financial incentives, system integration, and maintenance are addressed and provided. Improving equitable access requires more than making screening kits available. Tailored, culturally responsive, multi-faceted resources that could address perceived low bowel health literacy and cultural beliefs about screening, along with practitioners’ endorsement and encouragement, and enhanced patient navigation within primary care are perceived having potential benefits in supporting more equitable bowel cancer screening participation among underserved populations.

Contribution to Health Promotion

- Primary care can promote equitable bowel cancer screening through practitioner endorsement and patient navigation.
- The Alternative Access Model (AAM) may improve screening access for underserved, ethnically and socioeconomically diverse communities.
- Embedding bowel screening promotion and the AAM into existing preventive touchpoints, such as health assessments, could be an effective and sustainable model.
- Implementation of the primary care–based strategy and intervention requires attention to workflow, training, role clarity, technical support, and funding.
- Tailored multilingual, culturally responsive, and multi-faceted resources are needed to address bowel health literacy and screening concerns.

## Introduction

Australia has one of the highest colorectal cancer (CRC or bowel cancer) rates worldwide (Ferlay *et al*. 2024). Colorectal cancer was the fifth most common cancer in Australia, with an estimated 14 768 newly diagnosed cases in 2025. In the same year, it was the second most common cause of cancer-related death, with an age-standardized mortality rate estimated at 23 deaths per 100 000 people (Australian Institute of Health and Welfare 2026).

Regular participation in bowel cancer screening is one of the most effective interventions for reducing bowel cancer mortality and improving population health outcomes. Australia, like other high-income countries such as The Netherlands, Scotland, and Denmark (Flander *et al*. 2022), has a national-organized screening programme, the National Bowel Cancer Screening Program (NBCSP). Eligible Australians aged 50–74 years are sent a free faecal immunochemical test (FIT) kit by mail to their home every 2 years, while those aged 45–49 years can request a free kit from the programme or through their healthcare provider (HCP; Australian Government Department of Health Disability and Ageing 2026). Research from the Australian Institute of Health and Welfare (AIHW) shows that overall, people who take part in the programme are more likely to have precancerous lesions detected and removed; hence, stopping cancer from ever starting or bowel cancer detected earlier (using tools like colonoscopy and biopsy followed a positive screening result) when treatment is more effective and therefore, less likely to die from bowel cancer (AIHW 2018). Successful treatment rates at the localized stage reach as high as 90% (Chen *et al*. 2025, Cancer Council Australia [CCA] 2026). Boosting programme participation to 60% could save an estimated 83 000 lives by 2040 (Lew *et al*. 2017, CCA 2026).

Despite the effectiveness of screening, participation in Australia’s NBCSP remains low, and uptake is considerably lower among culturally and linguistically diverse (CALD) communities, a term commonly used in Australia to describe ethnic minority communities, one of the underserved populations. Participation rates are most recently calculated at 24.3%–31.1% among ethnic minority populations compared with 42% nationally (AIHW 2026). Existing evidence indicates that inequities in participation are driven by a combination of socioeconomic disadvantage, low bowel health literacy, and cultural factors (Phillipson *et al*. 2019). Evidence also highlights the importance of HCP endorsement and patient navigation as effective facilitators of bowel cancer screening participation, particularly among underserved populations, including ethnic minority communities (Lee *et al*. 2023, Mosquera *et al*. 2023).

Involvement of HCPs, particularly general practitioners (GPs), has been shown to improve bowel cancer screening participation (Dodd *et al*. 2019, Goodwin *et al*. 2019, Lofti-Jam *et al*. 2019, Tsipa *et al*. 2021) and has been reported as acceptable to patients (Brown *et al*. 2019, 2020, Goodwin *et al*. 2020). However, GPs in Australia have historically had limited direct involvement in the centralized NBCSP following its national implementation in 2006, because testing kits are mailed directly to eligible individuals. The centralized NBCSP eliminates the initial need to visit a doctor or clinic to source a kit, automatically invites eligible citizens every 2 years, and manages subsequent screening kit dispatches based on past participation.

Primary care clinicians have long been able to provide non-programme tests via a pathology request, thereby providing opportunistic support for screening. In order to utilize GP endorsement in a more systematic way, the Australian Government introduced the Alternative Access Model (AAM) in 2022, an alternative pathway and distribution framework aiming at enlisting trusted GPs to help remove participation barriers, particularly among under-screened minority populations. Under this model, primary care providers can bulk order, store, and distribute kits directly to eligible patients during routine consultations, thereby augmenting the traditional mail-out programme.

The mechanisms through which the AAM may improve screening participation, specifically HCP endorsement and patient navigation, are well supported by the empirical literature as strategies for addressing participation barriers (Hewitson *et al*. 2011, Goodwin *et al*. 2019, 2020). HCP endorsement refers to recommendations delivered by a trusted healthcare professional, such as a GP, encouraging engagement in a specific health behaviour. During face-to-face consultations, patient concerns related to fear, anxiety, shame, or poor understanding of bowel cancer and screening can be addressed (Lee *et al*. 2023). This form of direct communication has been shown to positively influence participation in bowel cancer screening by reinforcing recommendations and providing clear support from a trusted healthcare professional (Lee *et al*. 2023). Consistent with research involving stages of change models (Lee 2022, Myers *et al*. 2022), GP endorsement may provide motivation to participate in screening and support intention to act.

Patient navigation is a healthcare delivery intervention in which trained individuals assist patients to access healthcare services, follow specific care pathways, or enact specific health-related behaviours. In the context of bowel cancer screening, patient navigation supports individuals to complete screening tasks successfully and facilitates continuity across fragmented healthcare systems, reducing the likelihood of patients being lost to follow-up (Freeman 2013). Patient navigation may help patients move from intention to action by addressing knowledge, confidence, language, or system navigation barriers. Both HCP endorsement and patient navigation are particularly relevant for ethnic minority communities, where language barriers and medical mistrust can impede screening participation (Lee *et al*. 2023).

The AAM system depends on effective interaction between general practices and the National Cancer Screening Register (NCSR). Online digital channels enable HCPs to access the HCP Portal via Provider Digital Access (PRODA) and thereby interact with NCSR. The NCSR Hub, the digital software interface or widget that connects a clinic’s clinical software directly to the NCSR, lets providers view a patient’s screening history, check due dates and results, track follow-up alerts, and digitally submit forms—including recording when a kit is issued to a specific patient via the AAM or have a kit sent to the address held by the NCSR. While both the AAM and using NCSR Hub aim to increase HCPs’ involvement in NBCSP, the AAM works by letting a trusted clinician discuss the test and supply a physical kit immediately face-to-face, hence potentially overcoming barriers to participation.

The AAM has been previously trialled, between 2018 and 2019, in 44 practices supporting Indigenous Australians. Evaluation data indicated that Indigenous participants who received kits from a trusted health professional understood why the test was important and how to collect samples. Additionally, patient adherence to correct procedures was high; those who screened through the AAM did the test so much more promptly than usual pathway participants (Menzies School of Health Research 2020). The pilot also suggested that the costs of offering this alternative pathway were not high and that the adoption of the AAM as a permanent part of the NBCSP would help to address inequities in programme participation. Despite these promising findings from pilot implementation among Indigenous Australians, the AAM has not been widely adopted across primary care settings in Australia that serve the general population, in particular, ethnic minority groups/underserved populations. Reasons for this are not well understood, and research examining HCPs’ perspectives on the acceptability and feasibility of implementing the AAM within existing clinic operations, particularly clinics that provide service to people from minority populations, remains limited.

There is strong justification for improving acceptability and feasibility of the AAM given its potential to increase screening and address inequitable participation. In health systems, acceptability is how agreeable or appealing a proposed change is to patients, staff, and leaders. Feasibility is the extent to which that change can actually be done, built, or carried out in the real world using available time, money, and staff (Martin *et al*. 2026). In this study, acceptability was defined as whether primary care staff perceived the AAM positively, and feasibility as the extent to which the staff believed the AAM could be successfully and systematically implemented in the primary care setting and within current workflow.

Research has demonstrated that general practices and their staff, including practice nurses (PNs) and practice managers, are willing and well positioned to play a critical role in supporting cancer screening, increasing screening uptake, and improving health outcomes (Shi *et al*. 2002, Lee *et al*. 2021, Trankle *et al*. 2021, McIntosh *et al*. 2023). The extent to which these views are shared in practices that focus on health delivery to ethnic minority populations remains unknown. This study explores HCPs’ (who predominantly provide services to ethnic minority populations) views on the acceptability and feasibility of the implementation and the use of AAM, as well as strategies and resources needed to increase screening uptake and encourage participation in the AAM.

## Materials and methods

### Study design

Given the exploratory nature of this study, a qualitative design was adopted using semi-structured interviews to explore HCPs’ perceptions of their capacity to implement the AAM for bowel cancer screening within their clinics. Participants were eligible if they were GPs, PNs, or primarily practice managers (PMs), working within an accredited Australian general practice that has strong representation from ethnic minority groups. Two rounds of interviews were conducted ∼6 months apart.

### Participant recruitment and study setting

Participants were recruited using purposive sampling. In Phase 1, general practices with capacity and interest to participate in research were identified through the University of Melbourne’s Department of General Practice and Primary Care research database, Dynamics (Microsoft 2026). It was not possible to determine whether a clinic had implemented the AAM *a priori*, clinics were recruited by postcode to ensure representation across socioeconomically diverse areas with a high proportion of residents from ethnic minority backgrounds. Practices were initially contacted by telephone, with those expressing an interest in participating, then followed up by email with detailed study information. Participants from each practice were organized and confirmed by the practice managers, as usual.

The purpose of Phase 1 was to explore awareness of the AAM and its alignment with clinics’ current approaches to preventive health service and bowel cancer screening specifically. None of the seven clinics that participated in Phase 1 had the AAM implemented. The ‘National Bowel Cancer Screening Program—Quick read guide/Alternative access to kits model’ was shared with the AAM and was explained to Phase 1 participants.

The aim of Phase 2 was to find out whether any clinics that participated in Phase 1 implemented the AAM after Phase 1, and to explore perceived and actual challenges and enablers in implementation and the use of AAM. Phase 2 also intended to identify the support and resources needed to make implementation feasible. In Phase 2, follow-up purposive sampling was used to recruit the general practices and participants who had taken part in Phase 1. Two of the practices that participated in Phase 1 declined the invitation for Phase 2, and two new practices, one of them currently using the AAM, were recruited. Recruitment of and data collection from participants in both Phase 1 and Phase 2 continued until data saturation was considered to have been achieved. Participants received a gift voucher valued at AUD$75 upon completion of each interview in both phases.

Ethical approval for the study was obtained from the University of Melbourne Human Research Ethics Committee (Project ID 29485). All participants provided written informed consent prior to participation.

### Data collection

Data were collected through semi-structured interviews. Phase 1 interviews were conducted between June and October 2024 by two authors (J.J. and L.H.), and Phase 2 interviews were conducted between July and early September 2025 by a student researcher (E.Z.) and one senior researcher (J.J.).

All interviews (*n* = 37) were conducted in English. Thirty-four interviews took place in person at participating clinics at a date and time convenient to participants. Most interviews were conducted individually, except for two group interviews involving three staff members each (two GPs and one PN). Three interviews were conducted online via Zoom video conferencing. Prior to each interview, participants were provided with a Plain Language Statement and asked to provide written informed consent.

An interview schedule was developed to guide the interviews, and open-ended questions were used to encourage in-depth discussion. Although the interview schedule followed by a general sequence, the order and phrasing of questions were adapted dynamically in response to participants’ answers. Interviews were conducted in a conversational manner to elicit richer insights into participants’ perspectives (Braun and Clarke 2006).

In Phase 1, participants were asked about

- their awareness and understanding of the AAM, including whether clinics had implemented the AAM and why or why not and
- their perceived role and current involvement in promoting and supporting bowel cancer screening in general and the NBCSP specifically.

In Phase 2, participants were asked about

- perceived barriers and enablers to implementing and using the AAM, including ordering, storing, and distributing NBCSP screening kits; and
- suggestions for strategies to increase screening uptake and encouraging practice participation in the AAM.

All interview audio recordings were transcribed verbatim with Microsoft Word. Handwritten field notes were also taken during the interviews to describe contextual and non-verbal data.

### Data analysis

Interview transcripts were analysed using reflexive thematic analysis, following Braun and Clarke’s framework (Maguire and Delahunt 2017, Byrne 2022, Ahmed *et al*. 2025), and managed using NVivo 15 software. Data familiarization and initial inductive coding commenced as soon as transcripts from the first interview became available.

Initial coding of data collected from Phase 1 was undertaken independently by two researchers/authors (J.J. and L.H.), and data collected from Phase 2 was carried out independently by the student researcher (E.Z.) and one researcher (J.J.). The interview schedules were iteratively refined after each interview, and coding frameworks were iteratively built across phases. Interpretation of the open codes was discussed with other research team members, and appropriate changes were made.

Preliminary categories were independently generated by authors (J.J. and L.H.) in Phase 1 and authors (E.Z. and J.J.) in Phase 2, compared, reviewed, and discussed with the wider research team. Once consensus was reached and appropriate changes were made, the themes and sub-themes were further consolidated and named through a deliberative process. This process was repeated several times to maintain rigour.

## Results

Seven clinics and 18 HCPs participated in Phase 1, while seven clinics and 19 HCPs participated in Phase 2. Only one participating clinic in Phase 2 had implemented the AAM. Participants included PMs, PNs, and GPs working in clinics located across the state of Victoria, Australia. All participating clinics provided services to patients from ethnic minority communities. Participant characteristics: gender, HCP role, clinic location, as well as whether participants participated in Phase 1 or Phase 2 or both were presented in Table 1.

**Table 1:** Participant characteristics: gender, HCP role, and clinic location.

Characteristics	Number of participants (*n* = 37)	Participated in Phase 1	Participated in Phase 2	Participated in both Phase 1 and Phase 2
Gender				
Male	8	1	3	2
Female	29	7	6	8
Role				
General practitioner	13	1	4	4
Practice manager	9	3	2	2
Practice nurse	15	4	3	4
Clinic location (Victoria, Australia)				
Eastern	18	2	4	6
South-East	4	1	1	1
Inner-West	3	3		
North-West	5		3	1
South-West	2	2		
Western	1			1
Inner-northern	4			2

Four out of nine clinics are franchised, and 15 out of 37 interviewees are from franchised clinics.

### HCPs’ awareness of the AAM and alignment with current clinic practice

#### Awareness of the AAM

Nearly all participants in Phase 1 had little or no awareness of the AAM. Some were unable to clearly distinguish between distributing an NBCSP FIT kit obtained from the AAM and providing a kit supplied by a different pathology laboratory, mainly used for clinically indicated testing.

The alternative access? No, I don’t think I know… I did read something about it [the AAM] but I didn't think anything eventuated. (PM)

I think the difference from the pathology kits is that the AAM is part of the screening programme? So it's a different pathway. Is that right? Yeah, I probably haven't studied further on this. (GP)

Although awareness of the AAM was low, upon further discussion and explanation, participants came to view the model as a valuable initiative and were generally supportive.

I can see the appeal where the patient is automatically enrolled into the [NBCSP] system every two years … I was just going to say it is very time consuming to learn, probably some hands-on training [how to use the integrated system] would be good because just having that information [quick read guide—the AAM] available at my fingertips isn’t enough. (PM)

#### Views on participation in AAM training

Participants attributed the lack of awareness of the AAM to insufficient training and limited practical resources. For many participants, undergoing AAM-related training, even if linked to mandatory Continuing Professional Development (CPD), was not appealing. Participants described competing clinical priorities and differences in professional interest as reasons why training uptake would vary.

Say you have this [the AAM] as CPD training, I don’t think I would do it. It [selecting and attending trainings] depends on the doctor’s interests. (GP)

As a female young doctor, my patients are mainly young women. They come to see me mostly for sexual and reproductive health related conditions. I would be happy to do training about breast and/or cervical screening [rather than bowel cancer screening programme]. (GP)

Some participants suggested that training would be more attractive if it offered meaningful incentives. One participant noted that training linked to harder-to-obtain CPD categories, such as Measuring Outcomes, could encourage greater participation.

I don't know [if] training for CPD points is a way to catch everybody. Everybody’s different … One of the trickiest things to get points for is the audit called Measuring Outcomes. If your programme [implementation of the AAM] includes options to get these special points, lots of doctors will do it. (GP)

#### Current clinical practice in distributing screening kits

Providing pathology-supplied (non-NBCSP) bowel cancer testing kits was a common practice in clinics. Participants described this practice as convenient because the availability of kits allowed immediate action during consultations, particularly for patients with gastrointestinal symptoms, concerns, or risk factors such as family history.

I have [bowel cancer screening] kits given by various pathologies in my consultation room and will just give to my patient along with other routine tests when they are here. Patients can then drop their collected poo sample here in the clinic. (GP)

… if a patient has no symptoms, I recommend them to go to the Program. But if I refer them, basically they are clinically indicated … with a positive FIT result, the patient will be fast-tracked [for follow-up- colonoscopy]. (GP)

Participants who have been using the NCSR Hub (*n* = 6) described the recommended AAM implementation pathway as unnecessary. They suggested that interacting with Hub was an easier way of distributing an NBCSP kit and enrolling a patient into NCSR. For these participants, the AAM is not appealing.

There's something called the Hub, so I’ll show you. So this works better than the AAM, so we don’t need that one [the AAM] … I order kits for my patients, and the kits will be sent to their home … patients can come back to clinic after they receive kits if they need help for completing the test. (GP)

Staff from the only clinic that had the AAM implemented described increasing use of the model over time and highlighted the value of having a staff member explain the process to patients, including in the patient’s preferred language.

… we started with ordering 10 kits from the program, increased to 30, and now 100 kits … after the doctor see a patient, he will fill and print out the form … I then take patients through all steps [in the instruction sheet], and give them the instruction sheet in Vietnamese [the preferred/native language for the majority of our patients]. (PN/Admin staff)

A doctor from another clinic acknowledged the benefits of enrolling patients into the NBCSP via the AAM, including them receiving kits every 2 years as well as reminders from the programme, rather than relying only on clinic-based follow-up.

I think we can switch to the AAM. I didn’t realise [Unnamed Pathology provider, not the NBCSP] was a separate entity to the National Bowel Cancer [programme] … I think it’s in their (patients) best interest to get signed up for it [the NBCSP]. (GP)

### HCPs’ perception of their role and current involvement in the NBCSP

Participants described a broad sense of responsibility for their patients’ health and well-being. Many HCPs indicated that they attempted to raise preventive healthcare topics, including cancer screening, when opportunities arose during consultations. However, limited consultation time meant that preventive health promotion was often deferred until the end of the visit and could be difficult to introduce when patients had other immediate concerns.

You do your consultation when the patient comes in, they’ve got what they want to talk about first, for example, flu, stomach-ache, and then if you have a bit of time, you try to do these preventative health type of things after or they [patient] get upset. (GP)

Several GPs framed the NBCSP as primarily a government-led programme, particularly because invitation and kit distribution occur outside routine general practice workflows. At the same time, participants still saw a role for clinics in explaining the programme and supporting patients who asked questions or brought a kit into the practice.

… the government is doing that [sending a bowel screening kit], no? Why do we need to do the same thing? I will explain to my patients [the programme] if they ask, and staff in the front will go through details about completing a kit if patients bring in a received kit. (GP)

### HCPs’ perception of ethnic minority patients’ attitudes to the NBCSP

As a result of the purposive sampling adopted for recruitment of practices, many participants provided services to patients from ethnic minority backgrounds. Reported barriers to participation in bowel screening highlighted by the HCPs who supported patients from minority populations included unfamiliarity with population-based screening programme and the rationale for screening asymptomatic people using FITs, discomfort with the screening procedure described, poor access to culturally tailored information and resources, low general health literacy, limited knowledge of bowel health, and cultural beliefs. That conflicted with screening participation (e.g. cancer is incurable).

Participants reported that many ethnic minority patients who did not participate in bowel cancer screening had limited understanding of the Australian screening system and how it differed from healthcare pathways in their countries of birth. Those patients preferred to complete diagnostic procedures done overseas.

They [CALD patients] don’t understand screening programme. They don’t understand FITs—‘why can’t you just do colonoscopy’? So they don’t. They don’t have a concept of a screening programme. (GP)

Most of my patients prefer to go back to China and have endoscopy and colonoscopy done when they are there … and some other treatments such as dental treatment too. (GP)

Some participants stated that the stool collection process itself was unacceptable or unpleasant for some patients. These concerns related both to handling stool and to the perceived distastefulness of the overall process. Uncertainty about methods for accessing screening instruction in their preferred languages resulting lack of confidence in collecting appropriate samples was another concern.

They [CALD patients] don’t want to have anything to do with stool. Yeah, they think it’s dirty. … storing the collected first stool sample in the fridge really discourage lots of people [to do the test]. (GP)

There’s other people that find the whole process distasteful. Like, I can tell you my daughter did. She goes: Oh yuck, mum. (PM)

Yes, we know that the government has spent moneys and efforts to develop resources in different languages and they’re out there, but my patients don’t know where and how to get those materials … they can’t understand the invitation letter, the reminder/s and the results [as they are all in English]. (GP)

Some participants described cancer and death as difficult topics for them to discuss with their patients, in particular those from ethnic minority backgrounds, and those who considered themselves healthy.

… cancer screening, which links to cancer diagnosis, and potential death, is still very much a topic people from some cultural backgrounds want to avoid discussing … it’s also difficult to encourage and convince people who believe they are healthy and living a healthy lifestyle to screen. (GP)

Not the vast majority obviously [preferring to live happily without knowing], but I have a few, like, ignorance is bliss. (PN)

Despite these challenges, many participants described an ongoing commitment to educating patients, correcting misconceptions, and encouraging bowel cancer screening.

I think more and more people start to accept this [screening] … Yeah, with some explanation. You know, we discuss about the early diagnosis and early treatment and then they will accept. (GP)

The perceived value of practical explanation was also reflected in suggestions for demonstration kits. Participants felt that visual and hands-on resources could help clinicians explain the testing process more effectively than verbal instructions alone.

Well, if you have a demonstration kit that is available to doctors or nurses, it would be far more beneficial, than to just handing them a kit. (PM)

### Experienced and anticipated technology related challenges—software updates, system integration, and maintenance

Participants generally viewed updates of clinical information systems, i.e. a software currently used by a clinic, such as Best Practice and MedicalDirector as challenging. This problem was exacerbated by the AAM requirement to integrate the practice’s system with NCSR.

I can show you two [two researchers] the software [the clinic is using] … you see it takes forever to load … it gets worse after each update … I don’t know how this gonna work if we have the AAM implemented. (PN)

Participants anticipated that clinical structure, technical capacity and access to Information Technology (IT) support would shape feasibility of AAM adoption as well as its usability. Participants working at larger or franchised clinic structures reported that even when an IT specialist or team could support initial integration, ongoing maintenance and troubleshooting were challenging.

We got IT to help us with that [connecting current software with NCSR] and it’s not that easy to do it. I do think other clinics, if they don’t have a technology person/team, they will probably need more help with that for the initial implementation. (PM)

For staff working at smaller standalone clinics with fewer resources, initial integration appeared more daunting.

… we do have PRODA account … ideally the integration can be done by people who know technology … happy to order test kits from the NBCSP and store them in the clinic … I have no time to call 1800 number [support line for integration] for help … (PM)

Participants also shared other operation related issues, such as receiving notification of software changes without formal training, delayed information sharing and workflow change updates across clinics and less formal leadership support, which would have direct and/or indirect impact on the AAM implementation.

There was none [no formal training]. I don't think any doctor got any training. My husband is also a GP. So he told me this feature [using NCSR hub] is available and he didn't get any training from his practice either. We are just notified and it's up to the doctor to find out… you just kind of learn from your peers. (GP)

Usually every six weeks [for a staff meeting]. Every meeting has an agenda, and every doctor can add on to that [agenda]. So if there’s anything like we need to learn as a group, then we will just put it on the agenda … This is probably the job of a medical director. But we don’t have a medical director inside the clinic. (GP)

… I believed the practice manager has received information package from local PHN [Primary Health Networks (PHNs) are independent organizations that funded by the Australian Government to coordinate primary health care in their region. In total, Australia has 31 PHN regions which closely align with the state and territory local hospital networks. PHNs work closely with GP-led clinical councils—made up of doctors, nurses, allied and community health staff, specialists, and hospital management staff.] and placed it into my pigeonhole … if information can be shared and a brief training can be provided at the staff meeting [extra effort], it would work. (GP)

### Perceived challenges to routinely use the AAM

Participants identified several potential barriers to the routine use of the AAM. First and foremost, a longer than standard consultation (currently without a special Medicare code for it) as the model requires GPs to check a patient’s eligibility, discuss screening with the eligible patient, update and confirm the patient’s details, complete a form titled ‘Bowel Kit Issued by a Healthcare Provider’, give the completed form to the patient to sign before the patient seeing a nurse for practical support. Second, a clinic needs to regularly bulk order and store screening kits. This practice not only requires space but also dedicated staff to execute tasks such as paying attention to expiration dates of kits, checking inventory and so on.

Nearly all participants indicated that routine use would be more feasible if clinics had a clear structure, defined roles and responsibilities, and appropriate financial or workflow incentives.

The whole process requires a specific Medicare code … it definitely takes longer than a standard consultation … (GP)

… if it [the use of the AAM] translates directly to the thought of the [clinic’s] business … the GP clinic is a private entity … we need to make sure that we survive … (PM)

it’s [the use of the AAM] working for us because Dr Tran [pseudonym for a GP in the clinic] does everything before Van [pseudonym for a nurse in the clinic] explains all steps [of collecting samples] to patients in Vietnamese. We [the clinic] are paid for implementing and using the AAM. (PM)

### Suggested strategies for facilitating bowel cancer screening uptake and the use of the AAM in the primary care setting

#### Clearly define clinical roles and shared responsibilities

Leveraging the capabilities of the whole practice was identified as a key facilitator for the adoption of the AAM and increased screening uptake. Participants described how complementary staff roles could support the process. Specifically, GPs were seen as important for clinical endorsement of screening participation, and nurses were viewed as well placed to undertake patient navigation, i.e. explain the testing process and provide language support, whereas other staff, practice managers in particular, were responsible for inventory, ordering and storing kits, and coordinating practical implementation.

Our patients generally listen to doctors’ suggestions … if the screening thing is raised by the doctors, most patients are happily following and doing [the test]. (PM)

So, I don’t really want to be engaging with patients as to [ask] have they had a bowel cancer screening … my role is more about explaining to patients about the testing procedure in their native language/s and hand them the instructions sheet in Vietnamese. (PN)

#### Utilize other health check appointments for facilitation of screening participation

Most participants believed that embedding bowel cancer screening, including the use of the AAM, within preventive health checks, such as health assessments, would help overcome screening barriers. This approach enabled both GP endorsement and patient navigation and was viewed as especially logical for patients aged 45–49 because many clinics already conducted health assessments for this group and it has a Medicare rebate (a universal healthcare coverage).

at the last page of the [health] assessment template, it’s also a reminder for nurses to go through the cancer screening education to patient, just to let them [patients] know what the cancer screening services are available here in Australia that the patient can access. It’s all free. (PN)

Integrating bowel cancer screening promotion into annual or 6-monthly routine check-ups and chronic disease management plans was also viewed as a potential facilitator, particularly for cohorts already attending the clinic regularly for tests or ongoing care.

Most of our patients, in particular those from non-English speaking countries, come in for a whole blood test and some other specific tests every six months or every 12 months. I think including screening as one of the test items would work … of course if doctors are not entirely involved, it won’t work. (PN)

#### Access to relevant and tailored information about NBCSP

Participants emphasized the importance of accessible, tailored and useful education materials and resources, although they differed in their preferred dissemination methods. Some participants suggested that resources in GPs’ consultation rooms or waiting areas would be useful.

I think honestly having some resources in various languages in the waiting areas would be a great one, because they're sitting there waiting for the doctor. If it's in front of them, they'll see it, they'll pick it up and read it … I think having something that you can actually hand to the patient, they can take home with them, that’s probably the best. (PM)

it would be good if I have factsheets [e.g. colonoscopy v.s. kit screening] in my room and give it to my Chinese patients after consultations. (GP)

Other participants suggested that toilet-room posters in various language could be effective because they would remind patients about bowel screening in a context directly related to the test.

… bowel screening program … a poster in the toilet rooms would be good … they [patients] see it when using the toilet … it reminds them to check with doctors … it would work … (PN)

Participants also suggested waiting-area videos with captions in different languages and take-away brochures at the front desk. Overall, they favoured simple, short, tailored resources that were available in patients’ preferred languages and in multiple formats.

All in all, simple, short and relevant resources, ideally in language and in different formats, are useful for health promotion and promotion of screening program/s. (GP)

## Discussion

Despite poor initial knowledge and awareness of the AAM, the aim of facilitating patients’ engagement with the NBCSP, was well received by participants and implementation was perceived as feasible, but only if key challenges were addressed and enablers were utilized. Low awareness of the AAM and confusion about the difference between NBCSP and pathology-supplied screening kits were reported. There was also discussion of the relative advantage of NCSR Hub alone versus the AAM or the standard mail-out pathway. This confusion may influence whether eligible asymptomatic patients are enrolled in the NBCSP and receive programme-linked reminders and follow-up. Clear implementation guidance should therefore distinguish screening through the NBCSP from clinically indicated testing for symptomatic or higher-risk patients.

Technology and workflow integration were further barriers to adoption. Nearly all participants have experienced challenges related to software updates, and anticipated that the initial integration (PRODA requirements, access to HCP Portal, interaction with NCSR), and ongoing IT maintenance would be burdensome, particularly for smaller practices without dedicated technical support. Implementation support should therefore account for variable digital readiness across primary care and include streamlined onboarding, accessible technical assistance and minimal disruption to workflows as well as timely and efficient formal training. Those findings coincide with the recommendations highlighted in the pilot study (Menzies School of Health Research 2020). Here, it was noted that NBCSP is a programme that is operationally very complex and the alternative pathway (AAM) would work only if tailored and ongoing support was provided and a workable and sustainable system was developed and embedded into the health clinics’ routine practice.

Despite HCPs viewing preventive care as part of their professional role and primary care is well positioned to support screening participation, bowel screening promotion was still difficult to prioritize within time-restricted consultations. These findings confirmed the sentiment stated in the study carried out by Walker *et al*. (2025). Addressing this tension is important for screening promotion and the AAM implementation and use. The use of the AAM may be perceived by clinic staff as an additional administrative task unless it is integrated into routine workflows and supported by appropriate incentives. Embedding bowel screening promotion and the AAM into existing preventive touchpoints, such as health assessments, annual or 6-monthly check-ups, and chronic disease management appointments, may help normalize screening and reduce reliance on opportunistic end-of-consultation discussions.

The positive impact of practitioner endorsement, one of the key findings from the pilot implementation among Indigenous Australians (Menzies School of Health Research 2020), was confirmed as a recognized support for screening by the participants in our study. The finding of being able to provide culturally relevant and sensitive messaging about cancer and cancer screening to their patients, whether it is highlighted by participants from the AAM clinic or anticipated by participants from non-AAM clinics, aligns with evidence of the effectiveness of HCP endorsement—patients interpret the HCPs’ proactive involvement as a sign of importance and a call to act promptly (Zajac *et al*. 2010, Dawson *et al*. 2017, Brown *et al*. 2020). The experience shared by and effectiveness of the AAM clinic in terms of increased kit orders and distributions suggests that a well-established structure within which staff members take different responsibilities may be more sustainable than a GP-only model, particularly in clinics serving communities with additional navigation needs. Further research is required to identify whether appropriate use of the AAM in clinics with a high percentage of ethnic minority patients reduces inequity screening participation statistics. It is likely that effective implementation of the NBCSP requires intervention at the individual patient, community level and primary care providers (Kelly *et al*. 2026).

Findings obtained here emphasize that providing access to a kit (e.g. using the NCSR Hub) is not the same as ensuring meaningful access to screening participation. Participants described patient-level barriers, including low bowel health and bowel cancer literacy, discomfort with stool collection, avoidance of cancer-related discussions, fears of having cancer, unfamiliarity with screening programmes, and preference for colonoscopy or overseas care, as factors contributing to their patients’ low participation. These barriers are especially relevant for ethnic minority communities, where language, cultural beliefs, and differences between health systems may shape engagement (Christou *et al*. 2010, Phillipson *et al*. 2019, McEntee *et al*. 2022, Lee *et al*. 2023). There is a need to provide culturally and linguistically appropriate support to ‘push’ screening behaviour to completion (Freeman 2013).

Culturally and linguistically appropriate resources were viewed as important facilitators for promoting preventative health behaviour, bowel cancer screening specifically, as well AAM implementation. Resources identified as useful included waiting-room materials, posters in toilets, brochures, short videos, and take-home information in multiple languages. As Christou *et al*. (2010) highlighted, the content of educational resources requires tailoring to the needs and address-specific barriers of the ethnic minority populations supported by the practice. These resources may increase repeated exposure to screening messages but are unlikely to be sufficient on their own. Their greatest value is likely to occur when combined with practitioner endorsement and navigation, allowing patients to encounter screening information, discuss concerns with trusted staff, and receive practical support to complete the kit.

This study has several strengths. The qualitative design enabled in-depth exploration of perspectives from GPs, nurses, and practice managers across clinics serving ethnically diverse communities in the state of Victoria, Australia. This provided a whole-of-practice analysis of the AAM in general practice. The open-ended nature of the semi-structured interviews allowed participants to freely explain their thoughts without being curtailed by the interviewer, and the flexible follow-up questions prompted participants to examine further the nuances of the AAM in a practical setting.

However, the findings should be interpreted considering several limitations. The results reflect HCP perspectives and do not directly capture patient experiences of receiving an AAM kit or completing screening after practitioner endorsement as no patient data were collected; hence, the possible risk of deficit-based framing. In addition, only one participating clinic had implemented the AAM, meaning that many concerns about feasibility views were prospective rather than based on direct experience. Further limitations included the single-state setting, recruitment through a research-engaged practice database (Dynamic) with potential social desirability in provider accounts, and the fact that patient-level barriers are reported second-hand through provider perceptions.

This study explored HCPs’ acceptance of the AAM, an alternative pathway aimed at increasing uptake of the NBCSP through primary care, as well as their perception about the feasibility of rollout. This study also investigated the actual and potential importance of practitioner endorsement and patient navigation in promoting equitable access to the NBCSP; however, these concepts mostly reflect an interpretive synthesis of the data rather than discrete themes directly identified through analysis, as only one participating clinic had the AAM implemented. Future research should evaluate implementation outcomes across diverse practice settings, including adoption, fidelity, reach, patient acceptability, kit completion and follow-up after positive results, with particular attention to ethnic minority communities and other groups with lower screening participation.

## Conclusion

This study suggests that improving equitable access to bowel cancer screening in primary care requires more than making NBCSP kits available. Participants supported the aims of the AAM but identified time constraints, system integration and maintenance, lack of formal training, and incentives as key barriers to implementation. Perceived patients’ low health and bowel cancer literacy and specific cultural beliefs have a direct impact on reactions to messages about bowel screening and an indirect influence on the implementation and use of the AAM. A well-established structure with defined roles and responsibilities that leverage practitioner endorsement and patient navigation may strengthen the implementation by combining trusted GP recommendation with whole-of-practice support for education, kit distribution and patient navigation. To realize the potential equity benefits of the AAM, implementation should be supported by clear role delineation, funding of practical training, culturally appropriate and relevant resource development, investment in streamlined technology, and incentives that acknowledge the preventive health work required in general practice.

## Data Availability

The data that support the findings of this study are available on request from the corresponding author (J.J.). The data are not publicly available due to their containing information that could compromise the privacy of research participants.
